# Clinical Proteomics Reveals Vulnerabilities in Noninvasive Breast Ductal Carcinoma and Drives Personalized Treatment Strategies

**DOI:** 10.1158/2767-9764.CRC-24-0287

**Published:** 2025-01-23

**Authors:** Georgia Mitsa, Livia Florianova, Josiane Lafleur, Adriana Aguilar-Mahecha, Rene P. Zahedi, Sonia V. del Rincon, Mark Basik, Christoph H. Borchers, Gerald Batist

**Affiliations:** 1 Division of Experimental Medicine, McGill University, Montréal, Canada.; 2 Segal Cancer Proteomics Centre, Lady Davis Institute for Medical Research, Jewish General Hospital and McGill University, Montréal, Canada.; 3 Department of Pathology, McGill University, Montréal, Canada.; 4 Division of Pathology, Jewish General Hospital, Montréal, Canada.; 5 Segal Cancer Centre, Lady Davis Institute for Medical Research, Jewish General Hospital, Montréal, Canada.; 6 Manitoba Centre for Proteomics and Systems Biology, University of Manitoba, Winnipeg, Canada.; 7 Department of Internal Medicine, University of Manitoba, Winnipeg, Canada.; 8 Department of Biochemistry and Medical Genetics, University of Manitoba, Winnipeg, Canada.; 9 CancerCare Manitoba Research Institute, Winnipeg, Canada.; 10 Department of Medicine, McGill University, Montréal, Canada.; 11 Gerald Bronfman Department of Oncology, McGill University, Montréal, Canada.; 12 Department of Surgery, McGill University, Montréal, Canada.; 13 Exactis Innovation, Montréal, Canada.

## Abstract

Ductal carcinoma *in situ* (DCIS) is the most common type (80%) of noninvasive breast lesions in women. The lack of validated prognostic markers, limited patient numbers, and variable tissue quality have a significant impact on the diagnosis, risk stratification, patient enrollment, and results of clinical studies. In this study, we performed label-free quantitative proteomics on 50 clinical formalin-fixed, paraffin-embedded biopsies, validating 22 putative biomarkers from independent genetic studies. Our comprehensive proteomic phenotyping reveals more than 380 differentially expressed proteins and metabolic vulnerabilities, which can inform new therapeutic strategies for DCIS and invasive ductal carcinoma. Due to the readily druggable nature of proteins and metabolic enzymes or metabolism inhibitors, this study is of high interest for clinical research and the pharmaceutical industry. To further evaluate our findings, and to promote the clinical translation of our study, we developed a highly multiplexed targeted proteomics assay for 90 proteins associated with cancer metabolism, RNA regulation, and signature cancer pathways, such as PI3K/AKT/mTOR and EGFR/RAS/RAF.

**Significance:**

This study provides real-world evidence for DCIS, a disease for which currently no molecular tools or biomarkers exist, and gives an unbiased, comprehensive, and deep proteomic profile, identifying >380 actionable targets.

## Introduction

Ductal carcinoma *in situ* (DCIS) is a preinvasive (stage 0) neoplastic lesion that is associated with a ∼10-fold elevated risk of developing invasive breast cancer, e.g., invasive ductal carcinoma (IDC; ref. 1). Due to this increased risk, patients diagnosed with DCIS undergo aggressive treatment with breast-conserving surgery or total mastectomy with optional adjuvant therapy, i.e., radiotherapy or endocrine therapy.

Studies, however, show that if left untreated, only 20% to 50% of patients with DCIS will progress to IDC (2–5). This has led to global concerns about overtreatment of patients with DCIS, the resulting high economic burden for the healthcare system and, most importantly, a high psychologic burden for the patients. Tools and expression signatures to predict invasive progression for better informed clinical decision making are required, and many international trials are currently enrolling patients with DCIS for nonsurgical management by active surveillance, e.g., LORIS, LORD, and LARRIKIN, as described in Morrissey, and colleagues (6) The COMET trial (NCT02926911) in the United States is targeting histologically confirmed low-risk DCIS for a comparison of surgery to monitoring and endocrine therapy.

At present, the diagnosis of DCIS is based on calcifications observed during mammography screenings and histologic assessment of tissue biopsies, i.e., formalin-fixed and paraffin-embedded (FFPE) needle core biopsies. Five morphologic key features, high intratumor heterogeneity, poor interobserver agreement (7–10), and the lack of validated prognostic markers significantly impact clear diagnosis and risk stratification, as well as patient enrollment and the final results of clinical studies.

There is currently no precision oncology treatment available for patients diagnosed with DCIS. Postoperative (adjuvant) therapy is guided by IHC assays for estrogen and progesterone receptor status, HER2 expression status (by FISH), as well as BRCA1/2 mutation status. Clinical multigene assays, such as Oncotype DX/DCIS, MammaPrint, or PreludeDx DCIS, are sometimes used to clinically predict recurrence risks of patients but are not standard and only guide the use of adjuvant therapy.

Generally, DCIS studies are limited by patient number and tissue quality. Recent genomic landscaping studies on individual DCIS lesions identified putative biomarkers associated with progression toward IDC and gave insights into the underlying cancer biology. Multi-omics profiling of DCIS, however, is still challenging because DCIS and IDC lesions are mostly studied in FFPE-preserved samples; “pure” DCIS lesions can be very small in size as they are usually from minimally invasive needle core biopsies, and access to “pure” IDC lesions is limited, as most surgically removed IDC lesions also present *in situ* components and may follow effective neoadjuvant therapy.

**Figure 1:**
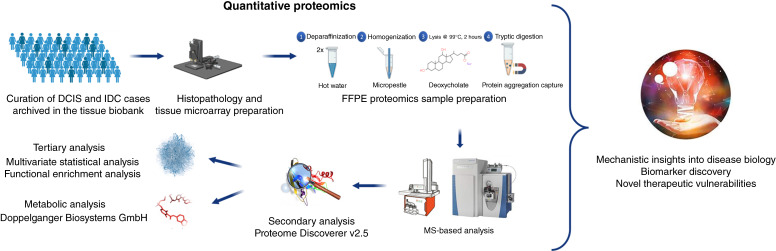
Experimental design. LFQ proteomics was performed in a cohort of carefully curated patients treated with DCIS and IDC (*n* = 51) to investigate changes in the protein expression. Protein extraction of FFPE tissue cores (1 mm diameter, ∼0.8 mm^3^ tissue volume) used an optimized FFPE proteomics protocol published in this study (11). The samples were analyzed on a “plug-and-play” platform built for standardization in clinical proteomics, and the data were processed using state-of-the-art data analysis tools, including machine learning/artificial intelligence–driven algorithms for improved and higher confidence mechanistic insights.

In this study, we made use of our recently published FFPE proteomics method that facilitates proteomic profiling on FFPE-preserved tissue cores (11). In a cohort of carefully curated patients treated with DCIS and IDC at the Segal Cancer Centre of the Jewish General Hospital (JGH) in Montreal (*n* = 51), we investigate changes in the protein expression of 29 “pure” DCIS lesions, 18 “pure” IDC lesions, 13 mixed-type lesions (IDC with DCIS components), and 9 cases in which DCIS and IDC are present in different lesions in the same patient, either synchronously or metachronously (see Fig. 1). (Note: “Metachronously” means that a DCIS case developed to IDC during clinical follow-up. “Synchronously” means that both DCIS and IDC lesions were collected at the same time, either on the same breast or the other breast.)

Data from recently published independent gene-expression studies investigating the progression from DCIS to IDC were used to complement the label-free protein expression data. Because FFPE preservation eliminates up to 85% of metabolites (12–16), we used Quantitative Systems Metabolism (QSM) technology from Doppelganger Biosystem GmbH, an artificial intelligence–driven metabolic analysis using proteomics data (17), for a comprehensive profiling of the central metabolism/energy metabolism. Guided by these results, we developed a highly multiplexed parallel reaction monitoring (PRM) assay for precise quantitation of 90 proteins that are associated with cancer metabolism, RNA regulation, and major cancer growth–associated pathways, such as PI3K/AKT/mTOR and EGFR/RAS/RAF.

## Materials and Methods

### Chemicals and reagents

All chemicals and reagents were purchased from Sigma-Aldrich unless otherwise specified. Sequencing-grade trypsin (Promega, P/N V511A) was used for the generation of tryptic peptides.

### Clinical specimens

Clinical specimens were obtained from patients who had provided written informed consent for the tissue biobanking part of the JGH Breast Biobank (protocol 05-006). The study was performed in accordance with the ethical standards laid down in the 1964 Declaration of Helsinki and was approved by the JGH Research Ethics Board.

A total of 50 clinical cases of patients diagnosed and treated with DCIS and/or IDC at the JGH were carefully curated by a pathologist with expertise in breast cancer to select lesions meeting the inclusion criteria for mass spectrometry (MS)-based analysis, i.e., at least 30% tumor cellularity and less than 10% necrosis. The patients were of Caucasian ethnicity ranging from 22 to 82 years of age at first diagnosis (median age 52 years). The patients were followed for a period of 1 to 18 years (median 8 years). During the period of follow-up, 43 patients had no evidence of disease, and 1 patient had metastatic disease, whereas 8 patients died of cancer. The cohort comprises 29 cases with “pure” DCIS lesions, 18 cases with “pure” IDC lesions, 13 cases with mixed-type lesions (IDC with DCIS components), and 9 cases with synchronous/metachronous DCIS and IDC. Clinical data for the patients are available in Supplementary Table S1.

### Sample preparation

One mm diameter tissue cores (∼0.8 mm^3^ tissue volume) were prepared from FFPE blocks enriching for DCIS- or IDC-only tumor cells. Excessive paraffin was trimmed off using a clean scalpel blade. Protein extraction was performed following our developed FFPE proteomics workflow for core needle biopsies. Briefly, paraffin was removed by incubation with hot water (∼80°C). Each deparaffinized core was mechanically disrupted using a micropestle (Sigma-Aldrich, #BAF199230001) in 250 μL of 2% sodium deoxycholate, 50 mmol/L Tris-HCl, and 10 mmol/L tris(2-carboxyethyl)phosphine, pH 8.5, followed by sequential incubation in Eppendorf ThermoMixer C for 20 minutes at 99°C (1,100 rpm) and for 2 hours at 80°C (1,100 rpm). Samples were cooled down on ice for 1 minute before a 15-minute centrifugation at 21,000 × *g* (4°C) to remove cell debris. The supernatant was collected into a Protein LoBinding tube (Eppendorf), and the total protein concentration was determined using Pierce Reducing Agent Compatible BCA Kit (Thermo Fisher Scientific, P/N 23252) following the manufacturer’s instructions. Free cysteine residues were alkylated with iodoacetamide to a final concentration of 30 mmol/L and incubated for 30 minutes at room temperature, protected from light.

For 2 μg of protein lysate, 2 μL of ferromagnetic beads with MagReSyn Hydroxyl functional groups (ReSyn Biosciences, 20 μg/mL) were equilibrated with 100 μL of 70% acetonitrile (ACN), briefly vortexed, and placed on a magnetic rack to remove the supernatant. This step was repeated another two times. Next, the protein extracts were added to the beads, and the sample was adjusted to a final concentration of 70% ACN, thoroughly vortexed, and incubated for 10 minutes at room temperature without shaking. The following washing steps were performed on a magnetic rack without disturbing the protein/bead aggregate. The supernatants were discarded, and the beads were washed on the magnetic rack with 1 mL of 95% ACN for 10 seconds, followed by a wash with 1 mL of 70% ACN without disturbing the protein/bead aggregate. The tubes were removed from the magnetic rack, 100 µL of digestion buffer [1:20 (w/w) trypsin:protein in 0.2 mol/L guanidine hydrochloride, 50 mmol/L ammonium bicarbonate, and 2 mmol/L CaCl_2_] were added, and the samples were incubated at 37°C for 12 hours. After acidification with trifluoroacetic acid to a final concentration of 2%, the tubes were placed on the magnetic rack for 1 minute, followed by removal of the supernatant. To remove residual beads, the samples were centrifuged at 20,000 × *g* for 10 minutes.

### Preparation of spiking solutions for the response curve and absolute quantitation

In order to promote translation of our findings and to validate label-free quantitation (LFQ) abundances with a more precise targeted MS approach, we developed a multiplexed PRM method to quantify 90 proteins in FFPE specimens, measuring the concentration of a unique signature peptide for each protein. All 90 peptides were measured in a single LC-MS/MS run. Two equimolar synthetic peptide mixtures (100 fmol/μg of each peptide) were prepared in 30% ACN with 0.1% formic acid in water (w/v); one mixture contained unlabeled peptides (light or NAT peptides), and the second mixture contained stable isotope-labeled standard peptides (heavy or SIS peptides). The light peptide mixture was used to develop the highly multiplexed PRM assay with optimized peptide-specific parameters, such as collision energy and charge state, whereas the heavy peptide mixture was used for normalization, serving as a spiking solution and internal standard for clinical samples.

Quantitation was performed using a seven-point response curve consisting of a variable amount of light peptides, ranging from 0.41 to 250 fmol (three orders of magnitude), and a constant amount of SIS peptides (50 fmol). Digested BSA (0.01 μg) was used as a surrogate matrix of the response curve. To determine the limit of detection (LOD), a double-blank sample was prepared. The blank sample consisted of 0.01 μg BSA digest spiked with 50 fmol of the SIS mixture and analyzed before and/or directly after the highest calibrant level of the response curve. For quantitation of endogenous protein in the patient samples, 50 fmol of SIS peptides were spiked into 1 μg total digested tissue protein, as determined by Pierce Reducing Agent Compatible BCA Kit.

### Data analysis

One μg of digested protein was preconcentrated on EV2001 C18 Evotips and separated on a heated (40°C) EV1137 column (15 cm × 150 μm, 1.5 μm particle size) using Evosep’s “extended method” (15 samples per day). The samples were analyzed by data-dependent acquisition mode on a Q Exactive Plus Orbitrap mass spectrometer operated with a Nanospray Flex ion source (both from Thermo Fisher Scientific) connected to an Evosep One high-performance liquid chromatography device (Evosep Biosystems). Full MS scans were acquired over the mass range from m/z 350 to m/z 1,500 at a resolution of 70,000 with an automatic gain control (AGC) target value of 1 × 10^6^ and a maximum injection time of 50 milliseconds. The 15 most intense precursor ions (charge states +2, +3, and +4) were isolated with a window of 1.2 Da and fragmented using a normalized collision energy of 28; the dynamic exclusion was set to 30 seconds. MS/MS spectra were acquired at a mass resolution of 17,500 using an AGC target value of 2 × 10^4^ and a maximum injection time of 64 milliseconds.

Chromatographic separation of all PRM runs was performed with the same equipment and buffers as described above. The Q Exactive Plus was operated in PRM mode at a resolution of 35,000. Target precursor ions were isolated with the quadrupole isolation window set to m/z 1.2. An AGC target of 3 × 10^6^ was used, allowing for a maximum injection time of 110 milliseconds. Data were acquired in time-scheduled mode, allowing a 2 minute retention time window for each target. Full MS scans were acquired in parallel at a low resolution (m/z 17,500) with an AGC target value of 1 × 10^6^ and a maximum injection time of 50 milliseconds to ensure sample quality.

MS data files are publicly available through the ProteomeXchange Consortium via the PRIDE partner repository (18) with the following dataset identifier: PXD040782. The synthetic peptides selected for this PRM assay were validated by others; information is available through the NCI’s Clinical Proteomic Tumor Analysis Consortium Assay Portal (assays.cancer.gov).

### Data processing and differential expression analysis

MS raw data were processed using Proteome Discoverer 2.5 (Thermo Fisher Scientific). Database searches were performed using SequestHT with multi-peptide search and a human Swiss-Prot database (January 2019; 20,414 target entries). LFQ was performed using the Minora Feature Detector node within Proteome Discoverer, and the Percolator software was used to calculate posterior error probabilities. Database searches were performed using trypsin as an enzyme with a maximum of two missed cleavages. Carbamidomethylation of cysteine (+57.021 Da) was set as a fixed modification, and oxidation of methionine (+15.995 Da) as variable modifications. Mass tolerances were set to 10 ppm for precursor ions and 0.02 Da for product ions. The data were filtered to a FDR <1% at the peptide and protein levels. Only proteins that were (i) identified with at least one protein unique peptide and (ii) quantified in ≥60% of replicates of at least one of the study groups were considered for the quantitative comparison. Protein LFQ data obtained from Proteome Discoverer were normalized based on summed protein intensities to correct for differences in sample loading. Missing protein intensity values were imputed using 1.5× the minimum observed intensity for this particular sample. The obtained normalized abundances were used for unpaired *t* tests (two tailed, 95% confidence interval) and differential expression analysis on log_2_-transformed data with multiple hypothesis testing using the Benjamini–Krieger false discovery approach (FDR 1%). Proteins having *q* values of <0.01 and absolute log_2_ fold changes (FC) >1 were considered differential between tested groups. Statistical analysis was performed using GraphPad Prism 9.

Raw PRM data were analyzed using Skyline (v22.2.0.351; ref. 19). Correct peak integration and visual verification of detected peaks was performed manually for each target, and the three to four highest and most stable transitions were selected for quantitation. A linear regression model with 1/*x*^2^ weighting using the SIS/NAT ratio of each target peptide was used for the calculation of concentrations. Only calibration levels meeting the following criteria were accepted for response curve generation and regression analysis; precision average <20% coefficient of variation per calibration level and accuracy average between 80% and 120% per calibrant level, quantified in at least three consecutive calibrant levels. The LOD describes the smallest concentration of the target peptide (analyte) that is likely to be reliably distinguished from instrument noise and at which detection is feasible. To determine the LOD, we use replicate injections from a double-blank sample, i.e., fixed concentration of the SIS peptides in the surrogate matrix. The average concentration of the double-blank plus 3.3× the SD of the blank replicates is used to calculate the lowest detectable concentration for each peptide. The limit of quantitation describes the lowest concentration at which the analyte can not only be reliably detected, but at which above mentioned precision and accuracy criteria are met. Here the limit of quantitation was defined as the lowest calibration level for each peptide. Proteins/peptides with more than 60% missing values were excluded from the downstream analysis.

### Functional enrichment analysis

Functional enrichment analysis was performed using the “Core Analysis” function within Ingenuity Pathway Analysis (Qiagen, Inc., content version: 81348237, release date: September 15, 2022; ref. 20). Ingenuity Knowledge Base was used as reference set, allowing direct and indirect relationships. Only molecules having expression *P* values <0.05 and absolute log_2_ FCs of >1 were considered for the core analysis. All other settings were kept with default parameters.

### Gene set enrichment analysis

A pre-ranked gene set enrichment analysis (GSEA) was performed using GSEA v4.3.2 (Broad Institute, Inc.) software. The gene list was ranked by differential expression using the SIGN function within Excel with calculated log_2_ FC and *P* value from an unpaired *t* test. A hallmark gene set Molecular Signature Database (MSigDB v2022.1; ref. 21) was used as reference gene set. The search allowed 1,000 permutations, with set sizes between 15 and 500 genes. Pathways were collapsed to remove redundancy and to increase selectivity and specificity. Data were visualized using the clusterProfiler (22) package within R.

### Metabolic analysis

Protein expression data from paired DCIS/IDC cases was sent to Doppelganger Biosystems Inc. for metabolic analysis using QSM technology (17).

### Data availability

MS data files are publicly available through the ProteomeXchange Consortium via the PRIDE partner repository (18) with the following dataset identifier: PXD040782. Clinical data are available in Supplementary Table S1. Hematoxylin and eosin images of clinical specimens used in this study are available via the dataset identifier on the PRIDE repository.

## Results

### DCIS and IDC are highly heterogeneous tumor phenotypes but build two distinct clusters in sparse partial least squares regression for discrimination analysis

**Figure 2:**
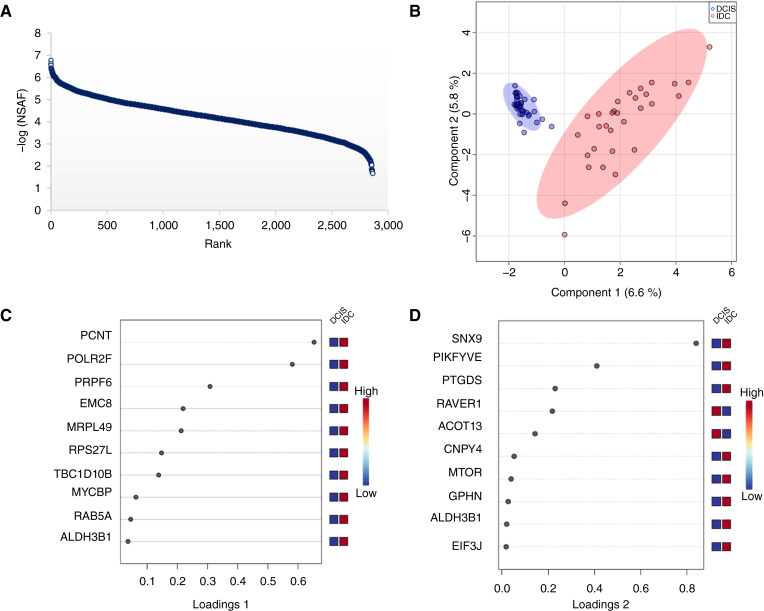
Data quality and evaluation of variability. **A,** Dynamic range of ∼2,860 proteins quantified in ductal breast cancer, at a 1% FDR. All −log_10_ values were based on NSAF values, which were used to normalize the spectral count (25). High NSAF values represent a high level of expression. Six orders of magnitude of the DCIS/IDC proteome are covered using ∼1% of the total sample and a standard data-dependent acquisition method without fractionation. **B,** Sparse partial least squares regression for discrimination analysis showing good clustering of the two study groups. The oval shape represents 95% confidence intervals. IQR and Robust regression and outlier removal methods identified no outlier samples. **C** and **D,** Loading plots of the sparse partial least squares regression for discrimination analysis showing proteins/genes that drive the variability and clustering between DCIS and IDC. The right *x*-axis shows expression levels of these drivers in the DCIS/IDC samples. NSAF, normalized spectral abundance factor.

Several genomic-centered studies have reported that both DCIS and IDC tumor phenotypes are highly heterogeneous (8–10, 23, 24), hampering clinical diagnosis but also limiting statistical power and robust assay development complementing clinical diagnosis. Using a streamlined FFPE proteomics workflow (11) with a standard label-free MS-based data analysis, we quantified more than 2,800 proteins at a 1% FDR on the protein and peptide levels. Using less than 1% of the total protein extracted from a single 1-mm FFPE tissue core, we cover six orders of magnitude of the DCIS/IDC proteome (Fig. 2A). Notably, the proteome of the two ductal breast cancer disease states seems to be clearly differential from each other, as a sparse partial least squares regression for discrimination analysis shows two distinct clusters between the study cohorts (Fig. 2B). The sparse partial least squares regression for discrimination analysis is a statistical method used for extracting and selecting important features from high-dimensional data to discriminate between different groups while simultaneously considering sparsity to improve interpretability and reduce overfitting (26). Based on the available clinical data (non-omics data) and small sample size, we are not in the position to infer any underlying patterns or biological relationships leading to this clustering on the protein level. Nevertheless, the top 10 features driving the proteomic variability between DCIS and IDC seem to reflect high transcriptional activity, extracellular matrix (ECM) remodeling, and inflammation processes (Fig. 2C and D).

### MS-based proteomics complements and supports independent genomic/transcriptomic studies of DCIS to IDC progression

Studies on the progression of DCIS to IDC have mainly used gene expression analysis or IHC/FISH on the protein level. The scientific community acknowledges misalignments between single-omics studies (27–30). We therefore compared MS-based label-free proteomics data with 49 differentially expressed genes identified by three recent larger-scale independent genomics/transcriptomics studies (9, 31, 32) and found 22 overlapping genes (see Table 1). Proteomics data identified gene products of Forkhead Box A1 (*FOXA1*), *POSTN*, *THBS2*, carbonic anhydrase 12 (*CA12*), *FN1*, and aldehyde dehydrogenase 1 (*ALDH1*) as differentially expressed proteins (DEP, unpaired *t* test; *P* < 0.05; see Supplementary Table S2).

**Table 1:** Overlapping molecules from independent gene expression studies and this proteomic profiling

UniProt ID	Gene symbol	IDC vs. DCIS	IDC mixed vs. DCIS mixed	IDC paired vs. DCIS paired	DCIS pure vs. DCIS mixed	IDC pure vs. IDC mixed
		*P* value	*P* value	*P* value	*P* value	*P* value
P55317	FOXA1	**<0.0001**	0.1584	na	0.0277	0.2796
Q9BV36	MLPH	0.9937	0.9549	0.1108	0.1053	0.2391
O43570	CA12	**<0.0001**	na	0.2684	0.9932	na
P02751	FN1	0.1223	*0.0584*	**0.0246**	0.3442	0.5544
P08123	COL1A2	0.2946	0.7485	0.2399	0.5725	0.6809
Q15063	POSTN	**0.006**	0.6849	**0.0293**	0.1870	0.3989
P35442	THBS2	**<0.0001**	0.3087	**0.0431**	0.5240	0.9132
Q02487	DSC3	0.2055	0.7676	0.4584	*0.0608*	na
P13647	KRT5	0.6350	0.6893	0.4811	**0.0008**	**0.0019**
P02533	KRT14	0.3695	0.195	0.7197	**0.0026**	**0.0006**
P04259	KRT6B	0.5053	na	0.5849	**0.0092**	**0.0491**
P19012	KRT15	0.0949	0.1307	0.3695	0.2278	0.5593
Q05682	CALD1	0.1707	0.9676	0.8580	0.1800	0.4176
P51884	LUM	0.2691	0.3142	0.8070	0.6572	0.5536
P46777	RPL5	0.7106	0.1105	0.1934	0.1819	0.0578
P05154	SERPINA5	0.1831	na	0.7365	0.4077	0.2000
P06401	PGR	0.6468	0.4329	0.1435	0.3075	0.3864
P04626	HER2	0.4177	0.2724	0.8663	0.3003	0.7259
P00403	COX2	0.2184	0.6032	0.1624	0.4270	0.1042
P00352	ALDH1	0.4049	0.4452	**0.0125**	0.3963	0.9840
P16070	CD44	0.3565	0.8431	0.1683	0.2674	0.9394
P06731	CEACAM5	*0.0592*	na	na	**0.0253**	na

Differential expression values of 22 proteins corresponding to genes proposed in the literature as biomarkers for DCIS to IDC progression. In bold, statistically significant entities with a student *t* test, *P* value <0.05; in italic, entities close to the set *P* value.

Abbreviation: na, not applicable; the protein was not quantified in that dataset.

The proteomics data show lower *FOXA1* expression in “pure” DCIS compared with “pure” IDC (*P* < 0.0001) and increased expression in mixed-type DCIS compared with “pure” DCIS (*P* = 0.03), suggesting a protective function of FOXA1. The loss or silencing of *FOXA1* observed in DCIS seems to promote cell migration and invasion. Interestingly, forced expression of *FOXA1* in MCF-7 (IDC cell line) inhibits growth and controls cell plasticity by repressing the basal-like phenotype (33, 34). Genetic studies associate *FOXA1* with heterochromatin remodeling, particularly affecting hormone receptor transcription (35) and regulation of the cell cycle with *BRCA1* (36, 37). Evidence of FOXA1 involvement in tumor progression on the (epi)genetic, transcriptomic, and proteomic levels warrants further investigation of FOXA1 as clinical biomarker and its clinical utility for DCIS risk stratification.

POSTN (periostin), THBS2 (thrombospondin 2), and FN1 (fibronectin) mediate cell–cell and cell–matrix interactions. POSTN, a downstream effector of β-catenin, activates PI3K/AKT and ERK pathways (38). In DCIS, these proteins have lower expression levels compared with IDC (*P* < 0.03, *P* < 0.04, *P* < 0.03, respectively), indicating stromal remodeling in DCIS to IDC progression.

*CA12* regulates the tumor microenvironment and metabolic pathways (39–41), with lower protein levels in “pure” DCIS compared with “pure” IDC (*P* < 0.0001). Loss of CA12 activity, which normally regulates pH levels, likely creates a more acidic environment, which can favor malignant cell survival and contribute to the progression from DCIS to IDC.

High *ALDH1* expression characterizes cancer stem cells associated with tumorigenesis, metastatic behavior, and poor outcomes (42, 43). Whereas an IHC-based profiling of DCIS did not associate ALDH1 with breast cancer events (9), our MS-based analysis on paired DCIS/IDC lesions does show a significantly higher concentration of ALDH1 in DCIS compared with IDC lesions (*P* = 0.01), supporting findings from stem cell biology that ALDH1 might be a functional and prognostic biomarker of tumorigenesis in DCIS.

Having access to “real-world” mixed-type lesions, the most prevalent clinical phenotype of breast ductal carcinoma, we were in the unique position to investigate the proteome of DCIS lesions that are likely active in the transition to IDC, depleted from intertumor heterogeneity. Comparing “pure” DCIS with mixed-type DCIS lesions revealed significantly lower protein levels of KRT5, KRT14, KRT6B, and CEACAM5 in “pure” DCIS lesions (*P* < 0.05; see Supplementary Table S3), indicating stromal remodeling as a key feature in the progression from precancer to invasive cancer, with prognostic value for DCIS management. High expression of keratines (KRTs) is linked to good prognosis in breast cancer, whereas lower levels are associated with invasive tumor proliferation (44–46). CEACAM5 (also CEA) expression has context-dependent impact and a protective function in breast cancer, with potential usefulness in disease monitoring (9, 47, 48). Similarly, comparing “pure” IDC with mixed-type IDC lesions showed a loss of KRT expression in mixed-type IDC (*P* < 0.05), suggesting a protective role of KRTs as a marker of progressiveness in DCIS.

HER2 protein overexpression constitutes a major prognostic and predictive marker in invasive breast carcinoma, and some recent studies indicate an association between HER2-positive DCIS with higher risk of local recurrence (49–52). However, there seems to be no substantial clinical impact so far, and our comprehensive proteomic profiling does not identify significant changes in HER2 expression between DCIS and IDC lesions (see Table 1).

### Loss of basal membrane stability, inflammatory processes, and epithelial-to-mesenchymal transition identified as key events driving DCIS progression

Having confirmed the results of genomic/transcriptomic studies in this setting using direct MS-based protein measurements, we turned to a global proteomics approach to discover further features of the DCIS–IDC scenario.

**Figure 3:**
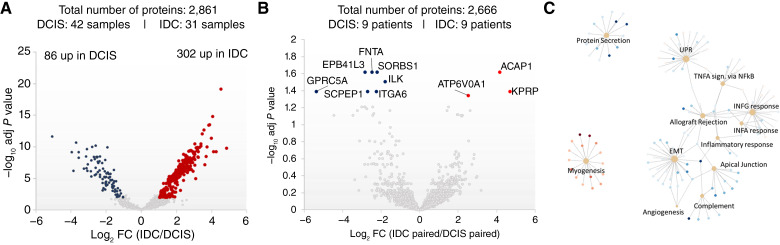
Differential expression analysis reflects loss of basal membrane stability, inflammatory processes, and epithelial–mesenchymal transition as key events toward DCIS to IDC progression. **A,** Volcano plot of the proteome of IDC compared with DCIS lesions showing 388 DEPs (unpaired *t* test with post hoc Benjamini–Krieger analysis *P* < 0.01, absolute log_2_ FC >2). **B,** Volcano plot of the proteome of paired IDC lesions compared with paired DCIS lesions showing 10 DEPs (unpaired *t* test with post hoc Benjamini–Krieger analysis *P* < 0.05, absolute log_2_ FC >2). **C,** Molecular networks representing up/downregulated pathways in IDC compared with DCIS lesions. adj, adjusted; EMT, epithelial-to-mesenchymal transition; UPR, unfolded protein response.

Differential expression analysis of more than 2,800 proteins identified in “pure” DCIS compared with IDC revealed ∼388 DEPs using an unpaired *t* test with the post hoc Benjamini–Krieger FDR method for multiple hypothesis testing (*q* < 0.01) and at least a 2-fold change in the protein expression between DCIS and IDC (Fig. 3A; Supplementary Table S2). To reduce interpatient variability, we compared proteomic profiles of DCIS and IDC lesions from the same patients (*n* = 9). Ten DEP were identified: ILK, ITGA4, GPRC5A, FNTA, SCPEP1, EPB41L3, and SORBS1, which were significantly more highly expressed in DCIS compared with IDC, whereas ACAP1, ATP6V0A1, and KPRP were significantly more highly expressed in IDC compared with DCIS (Fig. 3B; Supplementary Table S4).

ILK, an integrin-linked kinase, regulates integrin signaling and is associated with tumor growth and metastasis (53, 54). ITGA4 mediates cell–cell adhesions and is linked to cancer progression, inflammatory reactions, and ECM stemness (55–57). GPRC5A acts as an oncogene or tumor suppressor in different cancers (58–60). Androgen receptor–regulated FNTA enhances KRAS signaling and might be involved in tumorigenesis (61–66). SCPEP1 is associated with cancer development, growth, and metastasis (67–69). EPB41L3 is a tumor suppressor involved in apoptosis and cell-cycle regulation (70–73). Decreased expression in DCIS was observed for ATP6V0A1, which plays a role in pH homeostasis and tumor cell invasion (74–76). ACAP1 is associated with cell proliferation, migration, and immune infiltration in tumors (77–79). Loss of ACAP1 could indicate impaired immune response in IDC progression. KPRP, involved in keratinocyte differentiation (80, 81), might contribute to invasiveness when its expression is lost in DCIS.

## Discussion

Overall, proteomic profiling of DCIS identified more than 380 putative biomarkers (protein level) to clinically profile DCIS lesions for risk stratification and disease management. The association of the DEPs quantified in this study with hallmarks of cancer, such as remodeling of the tumor microenvironment (e.g., ILK, ITGA4, and SCPEP1), escape of apoptosis (e.g., ILK, GPRC5A, FNTA, and EPB41L3), deregulation of the apical junction and energy metabolism (e.g., ATP6V0A1, KPRP, and ITGA4), and inflammation and immune response processes (e.g., ACAP1 and ITGA4; Fig. 3C), warrants further investigation. Furthermore, most of the identified DEPs are readily druggable, and repurposing of FDA-approved anti-inflammatory drugs and antibiotics pose interesting treatment options for DCIS.

### EIF2 and PI3K/Akt/mTOR signaling pathways potentially drive IDC phenotype development through dysregulation of central energy metabolism in cancer

**Figure 4:**
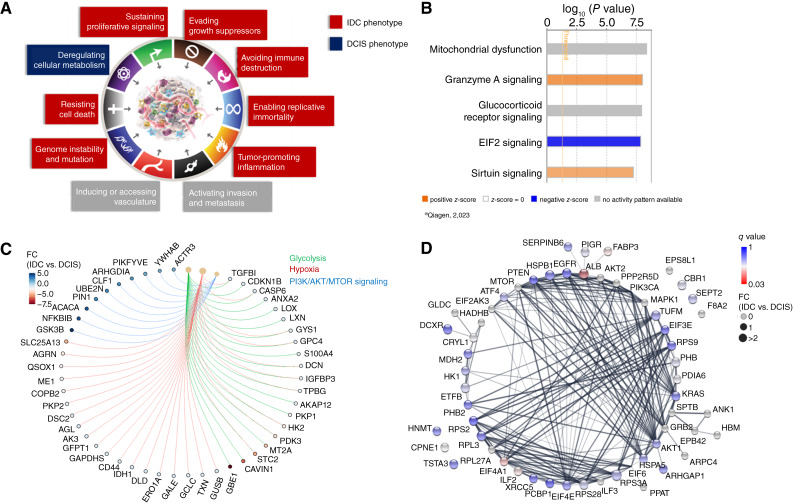
Dysregulation of central energy metabolism is a key event in the DCIS tumor phenotype. **A,** Graphical representation of hallmarks of cancer [modified from (82)] characteristic for proteomic tumor profiling of DCIS and IDC tumors. **B,** Top 5 canonical pathways from Ingenuity Pathway Analysis on DEPs in 42 DCIS and 31 IDC tumors. **C,** Signature proteins potentially driving DCIS progression through glycolysis, hypoxia (or “pseudohypoxia”), and PI3K/AKT/mTOR pathway, identified by GSEA. **D,** STRING network showing the protein expression profile of signature proteins associated with cancer metabolism, RNA regulation, and major cancer pathways, such as PI3K/AKT/mTOR and EGFR/RAS/RAF. Absolute concentration of the proteins was determined by PRM. The color of the nodes represents *q* values from multiple hypothesis testing using unpaired *t* tests with *post hoc* correction using the Benjamini–Krieger FDR method (1% FDR). The node size represents the FC. Gray nodes were not quantified, either because no SIS/NAT was available or because there were more than 60% missing values. Edges represent physical and/or functional interaction partners based on the STRING database.

A deeper look into the molecular relationships of all the DEPs we have identified by functional enrichment analysis and GSEA confirms the previously reported loss of basal layer integrity and epithelial to mesenchymal transitions as key events supporting IDC. Fig. 4A highlights cancer hallmarks that are predominant for the IDC and DCIS phenotypes, highlighting the dysregulation of cell metabolism as a key event in the DCIS phenotype. Proteomic profiling using MS-based techniques revealed metabolic vulnerabilities in DCIS that can provide insights into tumorigenic metabolic mechanisms that were missed by genomic/transcriptomic analysis alone.

Functional enrichment analysis using Ingenuity Pathway Analysis identifies mitochondrial dysfunction, granzyme A signaling, glucocorticoid receptor signaling, and sirtuin signaling as significantly enriched (*P* value of overlap <0.01) in our proteomics dataset, suggesting a dysregulation of glucose metabolism through a shift from oxidative phosphorylation (i.e., tricarboxylic acid cycle) to aerobic glycolysis (Fig. 4B and C; ref. 83).

Aerobic glycolysis is also known as the Warburg Effect and is characterized by high glucose uptake and glycolytic conversion of glucose to lactate to meet the high energy demands of proliferating cells (84). During glycolysis, glucose is converted to pyruvate. Cytosolic pyruvate can either enter the tricarboxylic acid cycle for oxidative phosphorylation and ATP production or be converted to lactate. Under normoxia, the metabolic fate of cytosolic pyruvate, and thus glucose metabolism, is regulated by pyruvate dehydrogenase complex (PDH) and lactate dehydrogenase, in which the PDH reaction is favored (84, 85), PI3K/AKT signaling can modulate the metabolic fate of pyruvate as an upstream regulator of PDH and lactate dehydrogenase, creating “pseudo-hypoxic” conditions that favor pyruvate conversion to lactate. The pivotal role of PI3K/AKT as an upstream regulator in metabolic reprogramming is comprehensively reviewed by Hoxhaj and colleagues (86) and involves the interaction with other proliferating signaling pathways, such as MAPK and mTOR. Our proteomic analysis of DCIS identified several differentially expressed molecules involved in glycolysis, hypoxia-mediated reactions, and PI3K/AKT/mTOR signaling (Fig. 4C) which warrant further investigation.

Metabolomic profiling of FFPE specimens is challenging, because ∼85% of metabolites are washed out during the preservation procedure. To nevertheless gain insights into metabolic changes occurring toward IDC progression, we conducted an artificial intelligence–based metabolic profiling using QSM technology, which is supported by more than 500 publications (17). Clear metabolic differences between DCIS/IDC lesions from the same patient (paired DCIS/IDC) were identified, but due to the large variability and small sample size (*n* = 9), metabolic differences between the groups were hard to assess. A multitude of functional markers with direct causal relation to ATP production capacity and utilization of glucose were nevertheless identified (Table 2). These findings confirm the dysregulation of energy metabolism toward IDC progression and suggest that the energy demand of transforming preinvasive cells (DCIS phenotype) is mainly achieved by fatty acid metabolism and lactate production.

**Table 2:** List of putative metabolic biomarkers identified by artificial intelligence–based metabolic profiling (inferred from proteomics data) of DCIS and IDC specimens from the same patient

ATP production capacity	CPT2, ACADM, HCDH, NDUBA, NDUBB, NDUV1, NDUV2, NDUS1, NDUS2, QCR1, QCR2, CY1, UCRI, QCR6, QCR7, QCR8, ATPA, ATPB, ATPD, ATP5H, ATP5I, ATPO, ADT3, MCEE, MUTA, THIK, THIM, ECHB, THIL, ODPA, ODPB, ODP2, DLDH, CISY, ACON, IDH3A, ODO2, SUCA, SUCB2, FUMH, MDHM, ACPM, and NDUA2
Glucose utilization	HXK1, ALDOC, PGAM1, and ENOG

To further evaluate and promote the translation of our findings into the clinic, we developed a highly multiplexed targeted MS assay for absolute quantitation of 90 signature peptides, associated with cancer metabolism, central energy metabolism, RNA regulation, and members of the PI3K/AKT/mTOR, EIF2, and EGFR/RAS/RAF signaling pathways. A complete list of peptides included in this assay is provided in Supplementary Table S5. The results of the PRM assay are depicted as STRING functional protein association network (Fig. 4D), in which the differential expression is represented by the node color and the absolute FC by the node size. These findings correlate well with the previously discussed observations from label-free proteomics and independent genomics/transcriptomics studies, showing that DCIS tumors have a tendency toward loss of metabolic functions. Albumin (ALB) is significantly higher expressed in the DCIS phenotype compared with the IDC phenotype (*q* value = 0.03). Studies associated low ALB levels with changes in the tumor microenvironment to more favorable conditions for disease progression and tumor migration, suggesting that serum ALB levels might have a prognostic value for cancer (87, 88). Other studies discuss ALB as a potent marker for inflammation and the nutritional status of patients, in which low ALB levels correlate with inflammatory processes resulting in higher morbidity and poor prognosis (89, 90). Our results support these findings and highlight remodeling of the tumor microenvironment, environmental stress (i.e., malnutrition, which inhibits EIF2 signaling; ref. 91), and inflammatory processes as key events toward IDC progression. It is, however, important to note that the amount of ALB observed in this study may be influenced by factors such as tissue perfusion, infiltration, or even the biopsy acquisition process itself. This makes it challenging to draw firm conclusions about its role in disease progression or to consider it as a reliable biomarker without further investigation into potential confounding variables.

In conclusion, clinical research on DCIS has been limited due to low sample numbers, high intertumor heterogeneity, and low tissue quality, as most DCIS lesions derive from diagnostic needle core biopsies and are FFPE. Although genetic/transcriptomic studies of DCIS progression provide a cellular blueprint of what might happen, genes cannot be readily targeted for therapy, and posttranslational modification cannot be assessed by genetic screening alone. Quantitative proteomics can complement and confirm genetic changes and provide a deeper look into the “real-life” tumor phenotype. The readily druggable nature of proteins makes quantitative proteomics studies attractive for clinical research. Additionally, MS-based studies allow both (i) discovery studies for comprehensive tumor profiling and (ii) validation studies in a highly multiplexed manner, with unprecedented accuracy, specificity, and sensitivity.

We established a LFQ proteomics pipeline suitable for needle core biopsy–sized FFPE specimens and performed a comprehensive proteomic phenotyping of DCIS and IDC using less than 1% of the total extracted protein material. We cover six orders of magnitude of the disease proteome and identify more than 380 DEPs that identify classical hallmarks of cancer, reflective for high transcriptional activity, ECM remodeling, and inflammation processes as key events toward IDC progression. We further identify dysregulation of glucose metabolism as a key event in the transition from preinvasive to invasive carcinoma. Guided by these results, we developed a highly multiplexed PRM assay for precise quantitation of 90 proteins that are associated with cancer metabolism, RNA regulation, and major cancer pathways, such as PI3K/AKT/mTOR and EGFR/RAS/RAF. We applied this assay to generate an activation profile of these signature proteins for proliferation and metabolic remodeling in cancer in “real-world” clinical samples and were able to support observations from label-free proteomics data with absolute concentrations in the mmol range, facilitating the translation of our findings into the clinic. Notably, proteomic profiling has revealed that FDA-approved drugs, such as antibiotics and NSAIDs, may be repurposed for DCIS and IDC treatment, as they have been shown to control and target proteins identified as key events toward IDC progression. The concept of repurposing antibiotics and NSAIDs has been a topic of investigation for several years (92, 93), and our proteomics data on DCIS-to-IDC progression support this concept.

It is important to highlight that this study design is applicable to many diseases with limited sample volumes and low tissue quality, as it requires only a fraction of the total sample amount, allowing discovery and validation studies in the same sample cohort. In our opinion, clinical proteomics is a versatile tool for comprehensive tumor phenotyping, able to capture a “real-life” snapshot of tumor phenotypes, representative of posttranslational modifications and epigenetic changes. More than 99% of published clinical biomarkers/genomic assays fail to enter clinical practice (94), but we show here that complementing genomics and transcriptomics studies with proteomics data, and *vice versa*, will help create a better understanding of underlying disease mechanisms and will better inform the selection of biomarker candidates and patient enrollment for clinical studies, ultimately improving the quality and final results of clinical trials.

This study provides real-world evidence data for DCIS, a disease for which currently no molecular tools or biomarkers exist, and gives an unbiased, comprehensive, and deep proteomic profile, identifying more than 380 actionable targets that can be taken further for functional analyses and biomarker analysis in a larger clinical cohort with more standardized and controlled sample collection, for example in a clinical trial.

## Supplementary Material

Supplementary Data Table T1 Anonymized Clinical Data

Supplementary Data Table T2 LFQ data DCIS vs IDC

Supplementary Data Table T3 LFQ data DCIS mixed vs IDC mixed

Supplementary Data Table T4 LFQ data DCIS paired vs IDC paired

Supplementary Data Table T5 PRM data DCIS vs IDC
